# Evaluation of an intervention aimed at supporting new parents: the *Baby Newsletter* project

**DOI:** 10.1186/s13052-020-00886-5

**Published:** 2020-09-04

**Authors:** Costantino Panza, Alessandro Volta, Serena Broccoli, Laura Bonvicini, Sally Kendall, Maddalena Marchesi, Paolo Giorgi Rossi

**Affiliations:** 1 Azienda USL-IRCCS di Reggio Emilia, Reggio Emilia, Italy; 2 Primary Care Service, Azienda USL-IRCCS di Reggio Emilia, Reggio Emilia, Italy; 3 Epidemiology Unit, Azienda USL-IRCCS di Reggio Emilia, Reggio Emilia, Italy; 4 Centre for Health Services Studies, University of Kent, Canterbury, UK

**Keywords:** Anticipatory guidance, Parenting, Self-efficacy, Parent-child relationship, Infant, Primary care

## Abstract

**Background:**

Anticipatory guidance for parents is commonly used to improve parenting skills. The objective of this pre/post-intervention controlled study was to evaluate the effectiveness of a periodic newsletter with advice on childcare and development in improving parenting self-efficacy.

**Methods:**

This was a non-randomized pre/post-intervention controlled study.

All the parents of children born between September 2014 and December 2015 resident in the S. Ilario d’Enza municipality (Italy) received eight Baby Newsletters. Parents resident in other municipalities of the same Health District were the control. Parents with linguistic barriers or with preterm or hospitalized children were excluded.

Improvement in parenting self-efficacy was measured through the TOPSE (Tool to Measure Parenting Self-Efficacy) questionnaire during the first week (t0) after delivery and at 5 (t1) and 12 months (t2) of life at two vaccination appointments. A score ranging from 0 to 60 was computed for each of the eight domains investigated by the TOPSE.

Variations of each TOPSE score between delivery and 12 months in the two groups were compared, adjusting for parity, education, age of parents, and child’s sex, and stratifying by parity and education.

**Results /findings:**

One hundred thirty-six families accepted to participate in the study. Scores at 12 months were higher than 1 week after delivery in both groups for all TOPSE domains. The improvement was slightly stronger in the Newsletter group for almost all the skills except *learning and knowledge* [difference in the mean of variation: -0.48 (95% CI: − 3.17; 2.21)]; the difference was significant only for *play and enjoyment* [2.18 (95% CI: 0.12; 4.25)]. The increase in scores in almost all domains was more pronounced for parents with high education level at first child.

**Conclusions:**

The intervention was effective in improving parents’ ability to play. However, it risks worsening existing differences between parents with high and with low education levels.

**Trial registration:**

Clinical trial registration: NCT03268408.

## Introduction

Parenting consists of a core set of interrelated components, including behaviors, attitudes, and knowledge regarding caregiving, stimulation, responsiveness, and safety [1]. Parenting is often defined as a primary mechanism of socialization [2]. Education and support for parents to learn and practice important parenting skills have the potential to promote supportive behaviors, parenting confidence, and the child’s well-being [3].

Many kinds of interventions supporting parents have been proposed based on ecological [4], transactional [5], attachment [6], or ecobiodevelopmental theory [7]. In pediatric primary care, the self-efficacy theoretical model is often applied [8]; it is based on parents’ belief that they are capable of being competent parents. Self-effective parents have confidence in their ability to successfully raise their child and are thus more convinced about what they do; this contributes to the quality of their relationship with their child [9, 10]. Parenting self-efficacy is usually assessed via self-report measures. The general measures focus on self-efficacy but also on other related constructs (competence, self-esteem, self-confidence, satisfaction, self-regulation) [11]. Among the 34 outcome measures identified by Wittkowski’s systematic review related to self-reported parenting skills, the “Tool to Measure Parenting Self-Efficacy” [12], developed by the UK National Health Service, focusses on 8 domains [13].

Anticipatory guidance consists of information that the clinician gives to the family on the stages of child development and growth and on the benefits of healthy practices, behaviors, and lifestyles. Further, it provides indications on how best to promote the child’s growth potential according to evidence-based medicine [14]. there are many anticipatory guidance topics, it is virtually impossible for a pediatrician to touch on them all during the short time of a well-child visit [15]. The Bright Futures Guidelines of the American Academy of Pediatrics have identified over 1200 anticipatory guidance that can be offered to parents during the 32 well-child visits (from ages 0 to 21 years), divided into 38 topics on 8 main themes: family support, child development, mental health, nutrition, physical activity, oral health, sexual development and sexuality, and accident prevention and safety [16]. Mass media, including printed matter such as newsletters, play a potentially important role in a comprehensive, population-based strategy to improve parents’ confidence, skills and knowledge concerning raising their child. Further, newsletters are an efficient and affordable format, in association with interviews with the pediatrician during well-child visits [17]. Effective anticipatory guidance must be timely, appropriate, and relevant so that key recommendations can be adopted by the family, thus making it formative in improving the parent’s and child’s well-being [16, 18–20]. There is evidence that some anticipatory guidance on injury prevention, feeding, vaccination, addiction, physical activity, abuse, infectious disease prevention and care, and reading aloud are effective [21–24].

Many surveys have highlighted how few parents receive anticipatory guidance [15, 25–31]. Parents with high education level have easier access to primary care, receive more advice, and ask the doctor for more information [25, 28], while families with less social support and who are more deprived (for example, newly arrived immigrants in Italy) often receive less advice and almost certainly have less access to understandable anticipatory guidance for developmental milestones [32]. Written advice is a useful tool for families [27] but may have some disadvantages related to parents’ literacy and health literacy [33, 34]. The use of pictograms and images and of short simple sentences can reduce this disadvantage [35, 36]. Patient information should be written at the reading level of a 9- or 10-year-old (4th–5th grade in the USA) [33], although even highly educated parents prefer succinct, easy-to-read materials [31].

In 2014, an intervention to improve parenting skills was implemented in one municipality in the province of Reggio Emilia, Northern Italy.

The intervention included mailing anticipatory guides, the Baby Newsletter, to all new parents. The Baby Newsletter was designed to prepare parents for the stages of their child’s development. It describes the child’s skills and abilities in relation to the correct moment of development and offers advice on what to do with the child at that specific age. As parents’ beliefs about child development serve as the foundation of their teaching and behavior management practices, this information helps parents not to have unrealistic expectations of their child’s skills, to improve the quality of their relationship, and to prevent possible behavior problems and stress on the family. Knowing about the domains of sensory, neuromotor, and socio-emotional development can help improve parents’ relationship with their child and the dyadic regulatory process [16, 37, 38]. In particular, the Newsletter offers simple activities such as word or singing games, reading, and music activities. Several studies have observed that these activities improve parental function, support intersubjectivity, reduce the duration of the baby’s crying, and improve regulation [39–43]. The aim of this study was to evaluate the effectiveness of the intervention in terms of parental self-efficacy as measured through the eight domains investigated by the TOPSE: *emotion and affection*; *play and enjoyment*; *empathy and understanding*; *pressures of parenting*; *self-acceptance*; *learning and knowledge*; *control*; *discipline and setting boundaries*.

## Methods

### Design

**Fig. 1:**
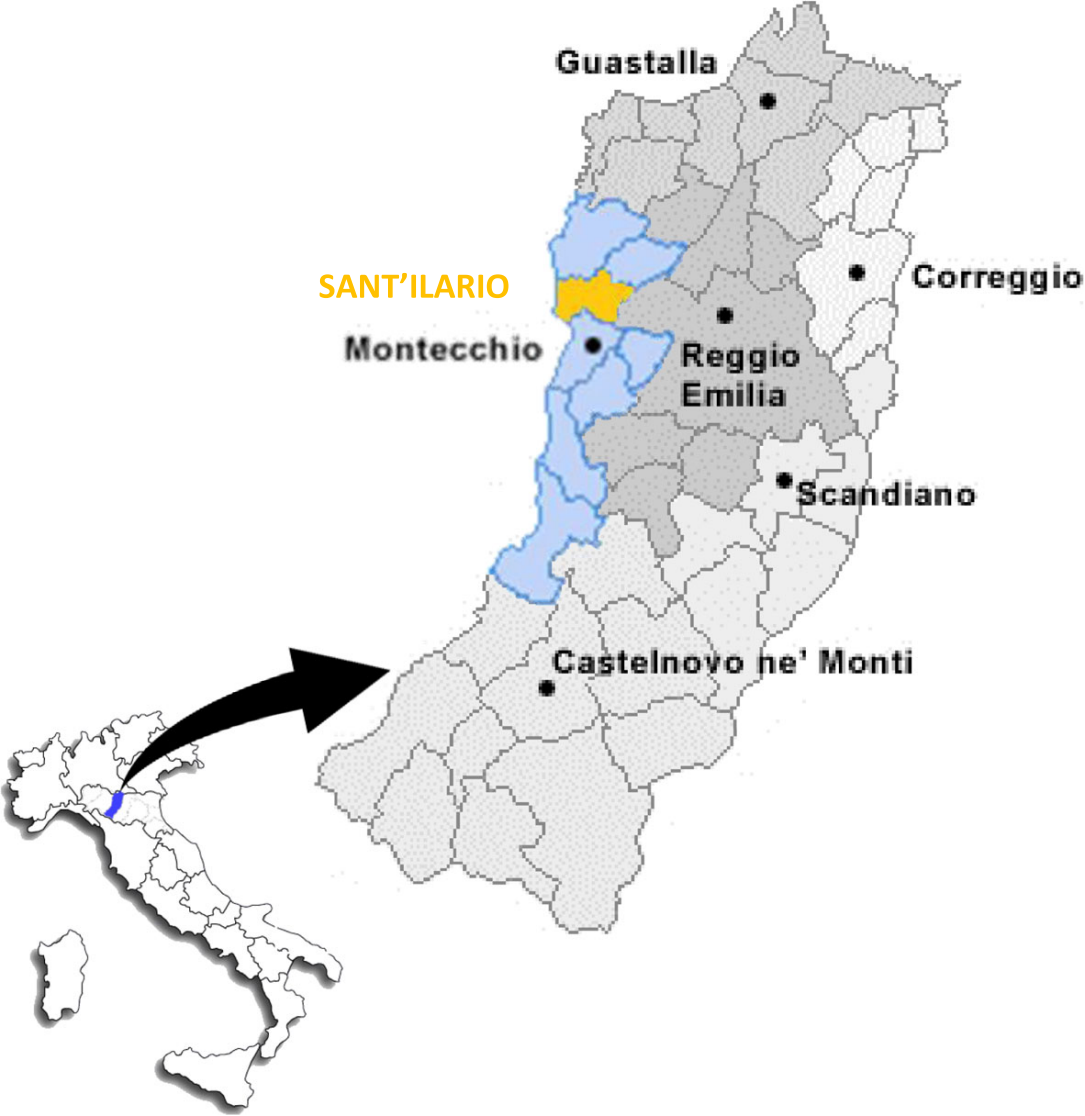
Reggio Emilia province. The intervention and the study area are highlighted

This was a non-randomized pre/post-intervention controlled study conducted in the Health District of Montecchio (62,800 inhabitants, 480 newborns per year), Reggio Emilia Province, Northern Italy [44] (Fig. 1). The evaluation study took place when the intervention had already started and was well established.

### Intervention

Eight newsletters were mailed to parents’ homes at child ages birth, 1 month, 2 months, 3 months, 4 months, 6 months, 8 months, and 10 months.

In the second- and fourth-month newsletters, the parents were also encouraged to borrow a book from the municipal library. This suggestion is in synergy with the national projects “Nati per Leggere – Nati per la Musica” [Born to read – born for music- www.natiperleggere.it/, www.natiperlamusica.org/], which are active throughout this Local Health Authority district. In this project, pediatricians give advice on singing and music and how to read to children, and parents are invited to participate in activities run by trained volunteers who teach the parents how to read and play with sounds with the baby.

The intervention started in January 2013 and involved all new parents resident in the S. Ilario d’Enza municipality, regardless of their participation in the evaluation study. The main contents of the newsletters concerned educational-pediatric contents [16] and community initiatives. A synoptic table (A3 format) was also provided, with a reminder of all the things “to do” with or for the child (0-3 m; 3-6 m; 6-9 m; 9-12 m). The anticipatory guidance was based on the most up-to-date scientific literature ([16, 28, 33, 45] (https://www.zerotothree.org/parenting, https://www.childwelfare.gov/topics/preventing/promoting/parenting/, https://www.publichealth.hscni.net/publications/birth-five, https://www.healthychildren.org/English/Pages/default.aspx)) and written in collaboration with Associazione Culturale Pediatri (ACP), a nonprofit Italian pediatric scientific and educational association, which reviewed the contents of each newsletter on the basis of the peer reviewed scientific literature. The ACP assumed editorial legal liability for the Baby Newsletter.

The contents were expressed in plain language to be comprehensible even to parents with low literacy. The comprehensibility of the Baby Newsletter was tested through a focus group made up of midwives and parents. We estimated that a reading level of an average 9–10-year-old (4th grade in the USA) was needed to understand the newsletter. Each newsletter page did not exceed 250–300 words (Additional files 1 and 2).

### Participants

Eligible participants were parents of children born between 01 September 2014 and 03 December 2015 at the Montecchio Hospital and resident in the Health District of Montecchio and all new parents in the same period resident in the municipality of S. Ilario d’Enza. Good comprehension of written Italian was inclusion criterion.

The exclusion criteria were delivering in facilities other than the Montecchio hospital (only for controls), parents of newborns transferred to another hospital immediately after birth for pathological conditions, and/ or parents of preterm newborns.

All the parents resident in the S. Ilario d’Enza municipality received the intervention (Baby Newsletter Group - BNG). Parents resident in other municipalities of the same Health District were the control (Control Group -CG).

### Data collection

Parents delivering at the Montecchio hospital were informed of the evaluation study before discharge by the operators of the outpatient pediatric service located at the hospital. Parents resident in S. Ilario d’Enza not delivering at the Montecchio hospital (about 33%) were asked to participate by the family pediatrician at his/her clinic during the first visit (4 pediatricians involved). If the parents agreed to participate, they signed an informed consent form and completed the baseline characteristic and habits questionnaire (one per family, collecting parents’ sociodemographic characteristics and basic knowledge about newborn care) and filled or arranged for the administration (one by the mother and one by the father) of TOPSE questionnaire (0–6 months version), no later than 15 days after birth (t0).

TOPSE questionnaire (complete version) was also administered to participants at the vaccination appointments at 5 (t1) and at 12 months (t2).

Participating parents who did not attend the vaccination visits were contacted by email, and then again by phone, to complete the questionnaire.

All children in the CG received 3 books at the end of the study as an acknowledgment of their participations. .

### Outcome definition

Parenting self-efficacy was measured through the TOPSE (Tool to Measure Parenting Self-Efficacy) questionnaire [13]. TOPSE has already been used to monitor parenting interventions [46]. It is structured in eight domains of six items each. The eight domains are *emotion and affection*, *play and enjoyment*, *empathy and understanding*, p*ressures of parenting*, *self-acceptance*, *learning and knowledge*, *control, and discipline and setting boundaries.* Each six-item domain is then summarized in a score ranging from 0 to 60. The questionnaire for 0–6 months does not include the sections on *control* or on *discipline and setting boundaries* since these are considered not relevant for this age group [13]. The Italian translation with back-translation was checked with the authors of the original version. A pilot with ten parents was conducted to check question clarity. A few issues were identified and corrected in agreement with the authors of the English version.

### Primary outcome measures

- Parenting score on the six scales of TOPSE questionnaire available at childbirth, delta (t2-t0);

### Secondary outcome measures

- Parenting score on each of the six scale of TOPSE questionnaire, delta (t1-t0);
- Parenting score on *control* and *discipline and setting boundaries* domains at 12 months.

### Sample size and study power

The study has six independent primary endpoints. No formal test of hypothesis was performed, *p*-values should be interpreted as continuous variables representing the probability that the observed difference or a larger one would occur under the hypothesis that the two groups had the same changes in a given parenting skill. No significance threshold was fixed. The study can be considered positive if the results of the six outcomes consistently go in the direction of an improvement. Given that there were fewer parents living in the area of the intervention, we adopted a 1:2 intervention:control ratio sampling. To detect a 5-point between-group difference in the delta (t2-t0) of one of the parenting scores with 5% type alpha error and power of 90%, assuming a standard deviation of 15, a sample size of 70 parents for the intervention group and of 140 for the control group was necessary, considering a 30% dropout rate at the one-year follow up.

### Data analyses

In order to assess external validity, a comparison between the study population and parents resident in the Montecchio district in the same period was made for education level and parity. This comparison was restricted to Italian mothers as foreigner mothers with language barriers were underrepresented in our sample.

To evaluate the internal validity, BNG and CG were compared by baseline characteristics, overall, and for those who filled the t2 questionnaires.

For TOPSE domains scores available at t0 and t2, main analyses were conducted according to difference-in-differences design: the two groups were compared for the delta (t2-t0) of the scores, and coefficients represent a comparison of mean of individual changes between groups.

Parents were considered as statistical units. The main analysis was adjusted for parity (first child yes/no), parent’s education level, and child’s sex. Linear regression models were built considering the intra-family correlation since we had data on both parents (in STATA software this is possible through the complex survey option, which computes the variance taking into account the intra-cluster correlation). R^2^ were reported for each model.

For the TOPSE domains *control* and *discipline and setting boundaries*, a regression at t2 adjusted for baseline characteristics (including other TOPSE domains that resulted unbalanced) was done as no baseline values were available for these scores.

Subgroup analyses are presented for mothers and fathers by parity (first child yes/no) and by parent’s education level (< 13, 13, > 13 years of school)).

Intention-to-treat analyses were conducted after checking that the family was included in the newsletter mailing list.

## Results

**Fig. 2:**
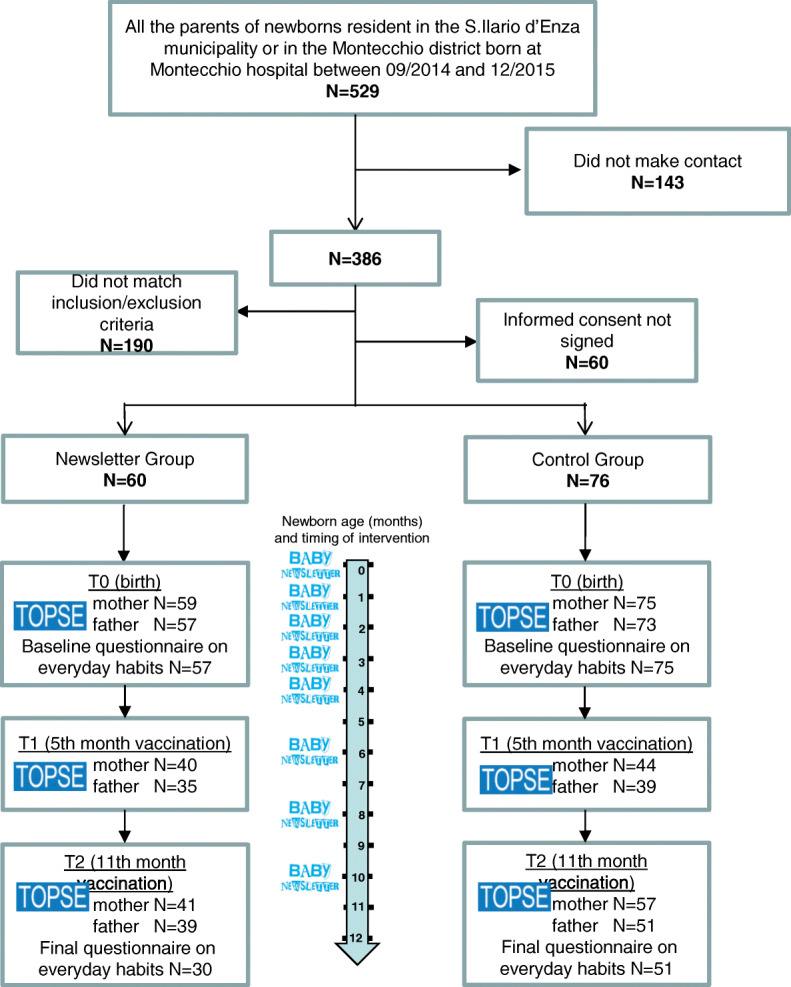
Flowchart of the study and timeframe for questionnaires and Baby Newsletter

Of the 529 new parents, 143 could not be reached within 15 days from birth, 190 were not eligible, and 60 refused to participate; 136 accepted to participate and completed the baseline questionnaire (25.7%) (Fig. 2).

Compared with resident parents in the area, the included parents were, as expected, less frequently foreigners, and the Italian parents were more educated. We compared the BNG and CG for distributions of sociodemographic variables collected in the baseline questionnaire, and differences were compatible with random fluctuations. (Table 1).

**Table 1:** Parents’ baseline socioeconomic characteristics, education, and smoking behavior by study group: the Baby Newsletter and the control group, with complete follow up

Characteristics		Newsletter group^1^		*with complete follow up*		Control group^2^		*with complete follow up*		Total		p-value(1 vs 2)
		N	m (sd)	*N*	*m (sd)*	N	m (sd)	*N*	*m (sd)*	N	m (sd)	
Child’s age when baseline questionnaire was completed (days)		57	8.0 (11.4)	*30*	*7.3 (9.7)*	72	2.6 (1.5)	*49*	*2.4 (1.4)*	129	5.0 (8.0)	< 0.01^+^
		N	%	***N***	***%***	N	%	***N***	***%***	N	%	
Who is completing the questionnaire	Mother	43	75.4	*22*	*73.3*	50	66.7	*32*	*62.7*	93	70.5	0.274*
	Father	14	24.6	*8*	*26.7*	25	33.3	*19*	*37.3*	39	29.5	
	Missing	0	0.0	*0*	*0.0*	0	0.0	*0*	*0.0*	0	0.0	
Home ownership	Yes	43	75.4	*25*	*83.3*	56	74.7	*39*	*76.5*	99	75.0	0.975*
	No	14	24.6	*5*	*16.7*	18	24.0	*11*	*21.6*	32	24.2	
	Missing	0	0.0	*0*	*0.0*	1	1.3	*1*	*2.0*	1	0.8	
Mother’s education level	Primary school	0	0.0	*0*	*0.0*	0	0.0	*0*	*0.0*	0	0.0	0.355*
	Medium school	9	15.8	*2*	*6.7*	9	12.0	*5*	*9.8*	18	13.6	
	High school	27	47.4	*12*	*40.0*	29	38.7	*19*	*37.3*	56	42.4	
	University	21	36.8	*16*	*53.3*	37	49.3	*27*	*52.9*	58	43.9	
	missing	0	0.0	*0*	*0.0*	0	0.0	*0*	*0.0*	0	0.0	
Father’s education level	Primary school	0	0.0	*0*	*0.0*	0	0.0	*0*	*0.0*	0	0.0	0.574*
	Medium school	12	21.1	*5*	*16.7*	11	14.7	*9*	*17.6*	23	17.4	
	High school	35	61.4	*20*	*66.7*	48	64.0	*29*	*56.9*	83	62.9	
	University	9	15.8	*5*	*16.7*	15	20.0	*12*	*23.5*	24	18.2	
	missing	1	1.8	*0*	*0.0*	1	1.3	*0*	*0.0*	2	1.5	
Mother’s profession	Worker	5	8.8	*3*	*10.0*	8	10.7	*5*	*9.8*	13	9.8	0.929^#^
	Employee	21	36.8	*11*	*36.7*	32	42.7	*22*	*43.1*	53	40.2	
	Freelance	7	12.3	*5*	*16.7*	6	8.0	*5*	*9.8*	13	9.8	
	Housewife	6	10.5	*2*	*6.7*	5	6.7	*3*	*5.9*	11	8.3	
	Unemployed	6	10.5	*2*	*6.7*	10	13.3	*8*	*15.7*	16	12.1	
	Manager	1	1.8	*0*	*0.0*	1	1.3	*1*	*2.0*	2	1.5	
	Other	9	15.8	*7*	*23.3*	13	17.3	*7*	*13.7*	22	16.7	
	missing	2	3.5	*0*	*0.0*	0	0.0	*0*	*0.0*	2	1.5	
Father’s profession	Worker	14	24.6	*8*	*26.7*	17	22.7	*11*	*21.6*	31	23.5	0.997^#^
	Employee	19	33.3	*11*	*36.7*	25	33.3	*19*	*37.3*	44	33.3	
	Artisan	5	8.8	*2*	*6.7*	7	9.3	*5*	*9.8*	12	9.1	
	Freelance	9	15.8	*4*	*13.3*	12	16.0	*6*	*11.8*	21	15.9	
	Unemployed	1	1.8	*0*	*0.0*	3	4.0	*1*	*2.0*	4	3.0	
	Manager	2	3.5	*1*	*3.3*	2	2.7	*2*	*3.9*	4	3.0	
	Other	7	12.3	*4*	*13.3*	8	10.7	*6*	*11.8*	15	11.4	
	missing	0	0.0	*0*	*0.0*	1	1.3	*1*	*2.0*	1	0.8	
Gross family income	Up to €36,152	33	57.9	*19*	*63.3*	43	57.3	*31*	*60.8*	76	57.6	0.905^#^
	From €36,153 to €70,000	21	36.8	*10*	*33.3*	29	38.7	*18*	*35.3*	50	37.9	
	From €70,001 to €100,000	1	1.8	*0*	*0.0*	2	2.7	*1*	*2.0*	3	2.3	
	Over € 100.000	2	3.5	*1*	*3.3*	1	1.3	*1*	*2.0*	3	2.3	
	missing	0	0.0	*0*	*0.0*	0	0.0	*0*	*0.0*	0	0.0	
Antenatal course	Yes	42	73.7	*21*	*70.0*	59	78.7	*41*	*80.4*	101	76.5	0.414*
	No	15	26.3	*9*	*30.0*	15	20.0	*9*	*17.6*	30	22.7	
	missing	0	0.0	*0*	*0.0*	1	1.3	*1*	*2.0*	1	0.8	
Other children	0	30	52.6	*18*	*60.0*	36	48.0	*26*	*51.0*	66	50.0	0.676^#^
	1	20	35.1	*8*	*26.7*	29	38.7	*20*	*39.2*	49	37.1	
	2	4	7.0	*1*	*3.3*	6	8.0	*2*	*3.9*	10	7.6	
	3	0	0.0	*0*	*0.0*	2	2.7	*1*	*2.0*	2	1.5	
	> 3	1	1.8	*1*	*3.3*	0	0.0	*0*	*0.0*	1	0.8	
	missing	2	3.5	*2*	*6.7*	2	2.7	*2*	*3.9*	4	3.0	
Smoking habit during pregnancy	Yes	4	7.0	*1*	*3.3*	2	2.7	*2*	*3.9*	6	4.5	0.404^#^
	No	52	91.2	*29*	*96.7*	69	92.0	*48*	*94.1*	121	91.7	
	missing	1	1.8	*0*	*0.0*	4	5.3	*1*	*2.0*	5	3.8	
Mother a smoker?	Yes	5	8.8	*1*	*3.3*	8	10.7	*5*	*9.8*	13	9.8	0.717*
	No	52	91.2	*29*	*96.7*	67	89.3	*46*	*90.2*	119	90.2	
	missing	0	0.0	*0*	*0.0*	0	0.0	*0*	*0.0*	0	0.0	
Father a smoker?	Yes	17	29.8	*6*	*20.0*	20	26.7	*15*	*29.4*	37	28.0	0.664*
	No	38	66.7	*23*	*76.7*	53	70.7	*35*	*68.6*	91	68.9	
	missing	2	3.5	*1*	*3.3*	2	2.7	*1*	*2.0*	4	3.0	
**Total**		**57**	**100.0**	***30***	**100.0**	**75**	**100.0**	***51***	**100.0**	**132**	**100.0**	

chi-square test(*) or Fisher’s exact test (^#^) or t-test (^+^) – Newsletter group vs control group, all regardless of the length of follow-up

The mean child’s age at baseline parent’s questionnaire was 8 days in the BNG group and 3 days in the control group (*p*-value < 0.01). Three baseline TOPSE questionnaires were completed more than 30 days after birth and were included in the analyses. All parents were contacted at t2; 53 and 68% in the BNG and CG, respectively, completed the final questionnaire on everyday habits. There were no differences between parents with complete follow up and all enrolled parents in both BNG and CG (Table 1). At the end of the follow up, 26 out of 29 families in the BNG reported having regularly received the newsletter, while 3, irregularly; 28 families declared they read all the received letters, 1 family declared it read only parts of the letters; 69% of the families retrieved the thank-you gift from the public library.

Completeness of TOPSE follow up was higher among mothers (72%) than fathers (67%).

Parenting skills at baseline were similar in the two groups for *emotion and affection* (delta − 0.2, *p*-value = 0.8827), *self-acceptance* (delta 0.8, p-value = 0.3753), and *play* (delta 1.8, p-value =0.2074), while the BNG had better scores for *pressures of parenting* (delta 5.3, p-value = < 0.001), *empathy and understanding* (delta 2.3, p-value = 0.0253), and *learning and knowledge* (delta 1.6, p-value = 0.0651) than the CG’s scores (Table 2). To test whether baseline differences were due to different timing in completing the questionnaire, an analysis was also conducted including only questionnaires completed in the first week after birth. However, the differences in TOPSE scores did not decrease.

**Table 2:** Baseline TOPSE parenting scores by study group: the Baby Newsletter and the control group, with TOPSE at 12 months. Scores are based on a six-item scale, each item has 0–10 values, and the total score ranges from 0 to 60

	Newsletter Group^1^			*with TOPSE at 12 months*			Control Group^2^			*with TOPSE at 12 months*			*p*-value t-test (1 vs2)
TOPSE domain at baseline	N	mean	95%CI	*N*	*mean*	*95%CI*	N	mean	95%CI	*N*	*mean*	*95%CI*	
Emotion	111	50.6	(49.2; 52.0)	*74*	*50.3*	*(48.7; 51.9)*	146	50.8	(49.8; 51.7)	*103*	*51.0*	*(49.9; 52.0)*	0.8827
Play	113	53.2	(51.9; 54.6)	*74*	*52.6*	*(50.8; 54.5)*	146	52.1	(51.0; 53.2)	*104*	*52.8*	*(51.6; 53.9)*	0.2074
Empathy	111	50.6	(49.1; 52.0)	*73*	*50.5*	*(48.7; 52.2)*	144	48.4	(47.1; 49.7)	*103*	*48.7*	*(47.2;50.1)*	0.0253
Pressures of parenting	110	47.9	(46.1; 49.7)	*73*	*47.9*	*(45.8; 50.1)*	141	42.6	(40.9; 44.3)	*100*	*42.8*	*(40.9; 44.7)*	< 0.001
Self-acceptance	108	51.8	(50.4; 53.1)	*73*	*51.2*	*(49.4; 52.9)*	140	51.0	(50.1; 52.0)	*99*	*51.7*	*(50.7; 52.7)*	0.3753
Learning	111	50.9	(49.7; 52.2)	*75*	*50.5*	*(49.0; 52.1)*	139	49.3	(48.2; 50.5)	*99*	*50.0*	*(48.7; 51.4)*	0.0651

**Fig. 3:**
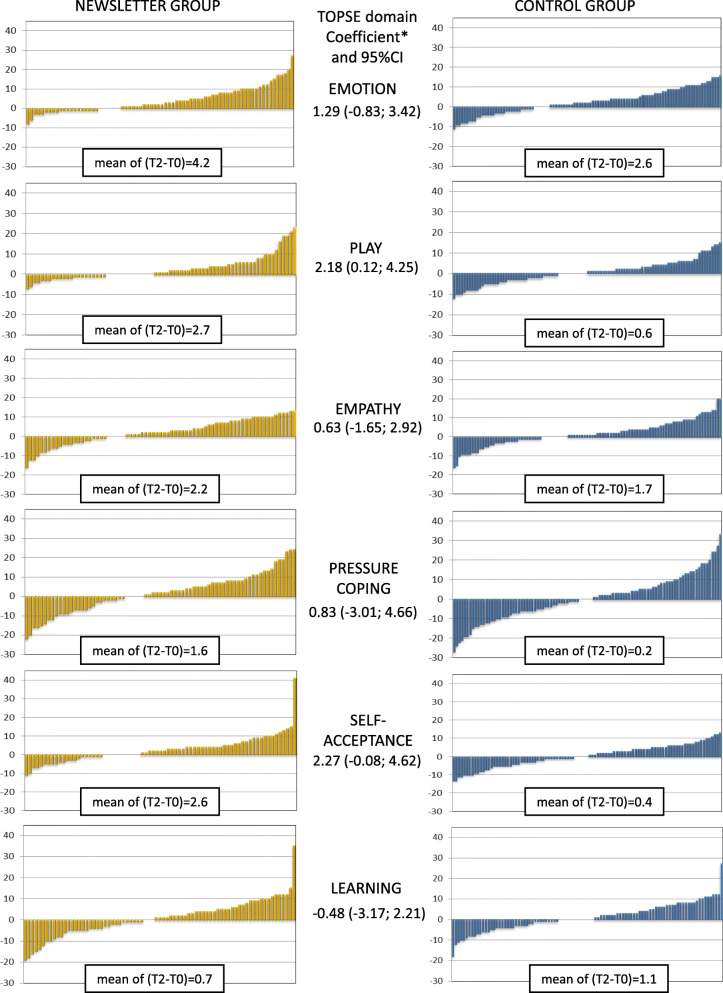
Distribution of differences between baseline (t0) and t2 values for parenting skills available in baseline questionnaires in the Baby Newsletter and in the control group. Scores are based on a six-item scale, each item has 0–10 values, and the total score ranges from 0 to 60. We also report mean value of the difference in within-groups and difference-in-differences with 95% confidence intervals. * Linear regression of delta score on group adjusted for child’s sex, parents’ age, parity, and education level. (R^2^: emotion = 0.0880, play = 0.0537, empathy = 0.0357, pressure coping = 0.0326, self-acceptance = 0.0379, learning = 0.0266)

Overall, parenting skill scores improved from t0 to t2 in all the domains. The improvement was slightly stronger in the BNG for almost all the skills except for *learning and knowledge* (linear regression coefficient − 0.48 [95%CI -3.17; 2.21]); differences were all compatible with random fluctuations except for *play and enjoyment* (Fig. 3) (linear regression coefficient 2.28 [95% CI 0.12; 4.25]). In all the models R2 are very small, the variation of the response variable that is explained by explanatory variables is very low. Results were similar for mothers and fathers (data not shown), while the differences in favor of the BNG were stronger in parents of firstborns, where all the coefficients were positive, particularly for *emotion and affection* and *self-acceptance* (linear regression coefficient 3.36 [95% CI 0.35; 6.37] and 4.55 [95% CI 1.01; 8.08], respectively). Differences were also stronger for parents with high education level. Indeed, for the *emotion and affection* and *pressures of parenting* domains we observed an opposite direction of the coefficient in parents with low education level, i.e. a greater improvement in the CG than in the BNG (Table 3): linear regression coefficients for parents with less than 13 years of school: -5.78 [95%CI -9.76; − 1.81] and − 12.55 [95%CI -22.57; − 2.52], respectively.

**Table 3:** Difference-in-differences (delta score) and 95% CI between groups adjusted for child’s sex, parents’ age: stratified analysis by education level and parity. Scores are based on a six-item scale, each item has 0–10 values, and the total score ranges from 0 to 60

TOPSE domain	Education Level				Parity		
	< 13 years	13 years	> 13 years	Interaction p-value	no other child	other children	Interaction p-value
Emotion*^##^	-5.78 (−9.76; −1.81)	1.60 (− 1.23; 4.43)	3.62 (0.64; 6.60)	0.0006	3.36 (0.35; 6.37)	− 1.27 (−4.10; 1.55)	0.0267
Play	−2.01 (−6.07; 2.05)	1.85 (− 0.72; 4.42)	4.47 (0.84; 8.10)	0.1225	2.39 (−0.16; 4.95)	1.88 (−1.59; 5.34)	0.8608
Empathy**	−0.27 (−4.60; 4.07)	−1.21 (− 4.61; 2.18)	3.96 (1.14; 6.79)	0.0265	1.44 (−1.89; 4.76)	− 0.26 (− 3.58; 3.06)	0.4645
Pressures of parenting **	− 12.55 (−22.57; − 2.52)	2.54 (− 2.77; 7.86)	1.70 (− 3.08; 6.48)	0.0127	2.75 (− 2.11; 7.61)	− 1.25 (− 8.17; 5.67)	0.3599
Self-acceptance^#^	1.22 (− 2.19; 4.63)	2.73 (− 0.91; 6.37)	2.04 (− 0.89; 4.98)	0.6751	4.55 (1.01; 8.08)	− 1.28 (−3.67; 1.09)	0.0093
Learning	−0.42 (− 6.19; 5.34)	−1.27 (−5.35; 2.81)	1.52 (− 1.82; 4.85)	0.3932	0.66 (− 3.41; 4.73)	−2.46 (− 5.20; 0.27)	0.2458

*p*-value interaction between groups and education level * < 0.01** < 0.05

*p*-value interaction between groups and parity ^#^ < 0.01 ^# #^ < 0.05

In the table only TOPSE domains evaluated at t0 and t2 are reported

**Fig. 4:**
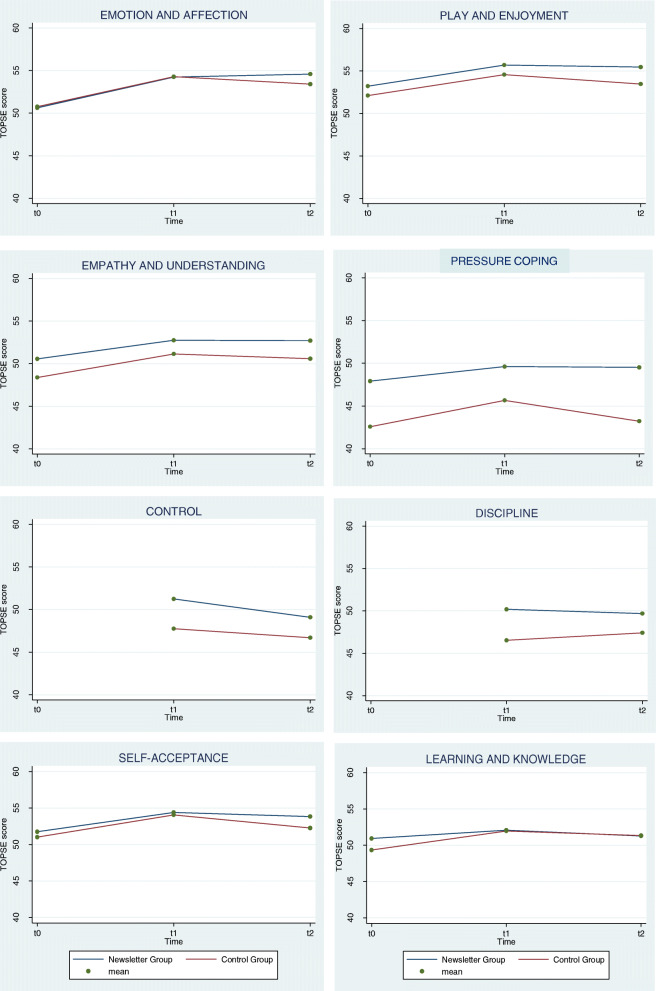
Mean score of TOPSE scale for treatment and time. Scores are based on a six-item scale, each item has 0–10 values, and the total score ranges from 0 to 60

Most of the changes from baseline occurred from t0 to t1, although the differences between the two groups became appreciable between t1 and t2 in *emotion and affection*, *play and enjoyment*, and *self-acceptance*, i.e., the three domains where the improvement was stronger in the BNG (Fig. 4).

For the two domains not available at t0, *control* and *setting boundaries*, we compared groups by t2 value. The results show higher scores for the BNG: linear regression coefficient for discipline and *setting boundaries* was 2.08 [95%CI -0.16; 4.32] R^2^ = 0.2102, and for *control*, 2.09 [95% CI -0.17; 4.35] R^2^ = 0.2526.

## Discussion

Overall parenting skill scores improved for all domains from birth to 12 months. The improvement was slightly stronger in the BNG for almost all the skills except *learning and knowledge* (on average, 0.48 points fewer on a scale from 0 to 60, 95% CI − 3.17 to 2.21), but the improvement was statistically significant only for *play and enjoyment* (on average, 2.18 points more on a scale from 0 to 60, 95% CI 0.12 to 4.25). Not surprisingly, differences were only perceptible in parents of firstborns [9, 47]. Education level also affected the results: there was an advantage for BNG only for the more educated parents in all outcomes.

Written advice interventions have been shown to be effective in causing positive health behavior changes [48] but there is little evidence on the effectiveness of written advice in improving parents’ skills and health behaviors in the care of their child in his/her first years of life [45, 49–51]. Parenting programs often are not built on a theoretical model [52]. Almost all pediatric parenting programs provide for focus groups, face-to-face sessions, and/ or advice offered in the clinical setting [52, 53] or provide home visits and group or individual sessions [9]. Few programs provide age-paced written advice sent by mail to support parenting [45, 49].

The intervention evaluated in this study, based on the parent’s self-efficacy, is affordable and sustainable. Furthermore, using email or mobile messaging apps may further reduce costs and possibly increase the spread of the intervention [49]; even if equity is a challange for technology-based parenting interventions [54, 55]. The Baby Newsletter allows all parents to receive written pediatric anticipatory guidance that would otherwise not be received and extends the pediatrician’s contact with the family [25, 56–58].

Finally, written guidelines issued by health authorities may play a fundamental role in the internet era, when free access to information is available to all without any verification of the source. The written messages in our newsletter are qualitatively reviewed and may help contrast poor quality information [31, 59, 60].

### Limitations and strengths

Because the study has a non-randomized design, we cannot rule out that differences in outcomes were due to differences in the enrolled populations in the control and the intervention arms.

Parents in the BNG all live in the same municipality, while those in the CG live in other surrounding municipalities. Even though there are no evident differences between the small towns in the study area and they have similar socioeconomic backgrounds, these towns still each may have some peculiarities, which could explain the observed differences, particularly at baseline. However, we did not find any differences in family composition or in parents’ socioeconomic characteristics; the only difference was the timing of baseline questionnaire. In the BNG, some parents, who did not deliver in the Montecchio hospital, were interviewed after hospital discharge, leading to a greater delay in the intervention group than in the CG. This difference in the timing of the baseline assessment, however, does not explain the differences in the TOPSE score at time 0 that we observed for *empathy* and *pressures of parenting*. In fact, even limiting the analysis to only questionnaires completed in the first week since delivery, the differences were not reduced at all Nevertheless, if the better baseline scores were due to delayed interviews, we would expect a smaller increase in the BNG. Furthermore, although family pediatricians do not have a predefined catchment area, the same four pediatricians provide care for most of the children in the BNG; those in the CG, instead, are cared for by about 10 pediatricians, including the four caring for the majority of the BNG. Nevertheless, the difference-in-differences design should partially address the issue of the lack of randomization.

The main disadvantage of the non-randomized controlled trial study design is that we cannot control for unmeasured or unknown confounding variables. However, this quasi-experimental design is very useful in evaluating community-based interventions like ours. In fact, conducting an RCT often requires an experimental setting at a few, highly selected sites, often needing to slightly modify the intervention to be adapted to the randomized design.

Another limitation is the use of a questionnaire that has been validated in English [46] but not in Italian. However, the observed results are consistent with each other and with most theoretical assumptions. The increase in parenting skills in the child’s first year of life was expected and confirms that the adopted questionnaire was able to capture the expected changes.

It is striking that the differences were noticeable only in parents of firstborns, as expected for an intervention aimed at informing parents about what is going to happen to their child in the upcoming weeks. Further, the differences in effectiveness of the intervention in terms of the parents’ education level were consistent with other studies on interventions aimed at changing behaviors [61], but also confirmed our a priori concern about scarce effectiveness of the intervention in parents with low literacy. On the other hand, these observations support the idea that some of the observed differences between the two groups were causally linked with the intervention.

The offer of new universal services is recognized to be effectively used by wealthy families and scarcely employed in low-income families, even if they achieve the expected benefits too, in the end [62, 63]. To improve the use of the Babynewsletter in the vulnerable families, in the future, family pediatricians may devote more attention to the discussion of anticipatory guidance during the well-child visits with low literacy parents and focus groups may be provided upon invitation for families with fewer resources. In this way the babynewsletter can progress from a general universalistic intervention to a proportionate universalistic intervention and therefore reduce the possible inequities that universalistic intervention can create [64].

Measurements are related to the parents’ self-confidence and self-efficacy; parents’ actual behaviors or outcomes on their children were not measured. A critical limitation of written guidance is parental literacy and health literacy. For this reason, it is considered necessary that written advice be at the fourth grade reading level [33]. Reading problems or language barriers are a challenge for some parents [65], who may feel judged in their reading abilities, thus increasing stress.

A strength of this study is the involvement of fathers, who are rarely included in studies on interventions to promote parenting [66].

## Conclusion

Written guidance on child development and on activities that parents can do with their child in the first year of life is associated with an increase in the parents’ self-confidence and self-efficacy in playing with the child and enjoying parenthood. In the subgroup with a low education level, however, there is an increase in stress and low control of emotions. Anticipatory guidance, even when designed for parents with a low education level, could have adverse effects in those parents, making equity the main challenge in interventions to improve parenting skills.

## Supplementary information

**Additional file 1.**

**Additional file 2.**

## Data Availability

The datasets analysed during the current study are available from the corresponding author on reasonable request.

## Abbreviations

TOPSE: Tool to measure parenting self-efficacy
BNG: Baby newsletter group
CG: Control group
t0: time at enrolment, when TOPSE questionnaire was completed first (2 to 15 days after birth)
t1: first follow up, when TOPSE questionnaire was completed again (about 5 months after birth)
t2: second and last follow up, when questionnaire was completed for third time (about 12 months after birth)
