# Supplementary material for: Evaluation of an intervention aimed at supporting new parents: the Baby Newsletter project
Source: Ital J Pediatr. 2020 Sep 4;46:123. doi: 10.1186/s13052-020-00886-5 (PMC7487811; doi:10.1186/s13052-020-00886-5)
Supplement: Supplementary file 1 — Additional file 1. [file 13052_2020_886_MOESM1_ESM.zip › Appendix 1_first month.pdf]

COSA SIGNIFICA ESSERE UN NEONATO

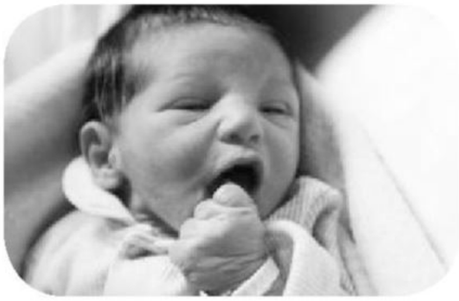

Ho bisogno che qualcuno mi ami, mi nutra, mi tenga in braccio e giochi con me.

Mi piace sentire il calore, ma non troppo caldo. Attenzione a questi segnali: sudorazione, capelli bagnati, guance arrossate, respiro veloce.

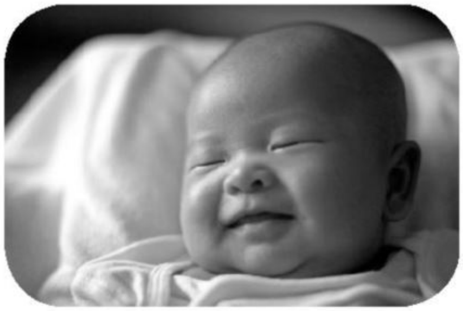

Non mi piace il baccano/frastuono.

Mi piace essere sostenuto con delicatezza e gentilezza

Mi piace stare molto a contatto con chi mi vuole bene.

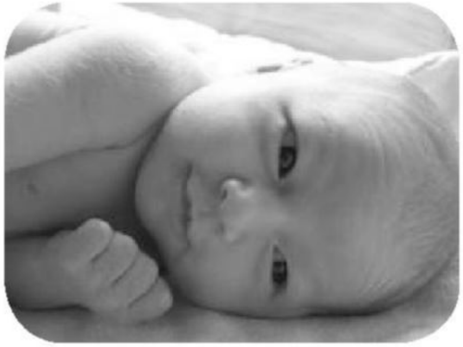

Mi piace dormire.

Spesso sono affamato, anche ogni 1-2 ore.

Posso essere irritato e posso piangere tanto.

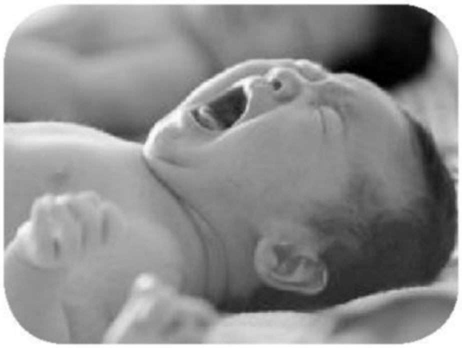

La mia testa è grande e le mie braccia e gambe sono corte; quando mi abbracci, metti il tuo braccio sotto al mio collo per aiutarmi a reggere la testa.

Io tengo braccia e gambe flesse/rannicchiate e le mie mani sono chiuse a pugno.
La mia pelle si può squamare.
