# Supplementary figures and images for: Evaluation of an intervention aimed at supporting new parents: the Baby Newsletter project
Source: Ital J Pediatr. 2020 Sep 4;46:123. doi: 10.1186/s13052-020-00886-5 (PMC7487811; doi:10.1186/s13052-020-00886-5)

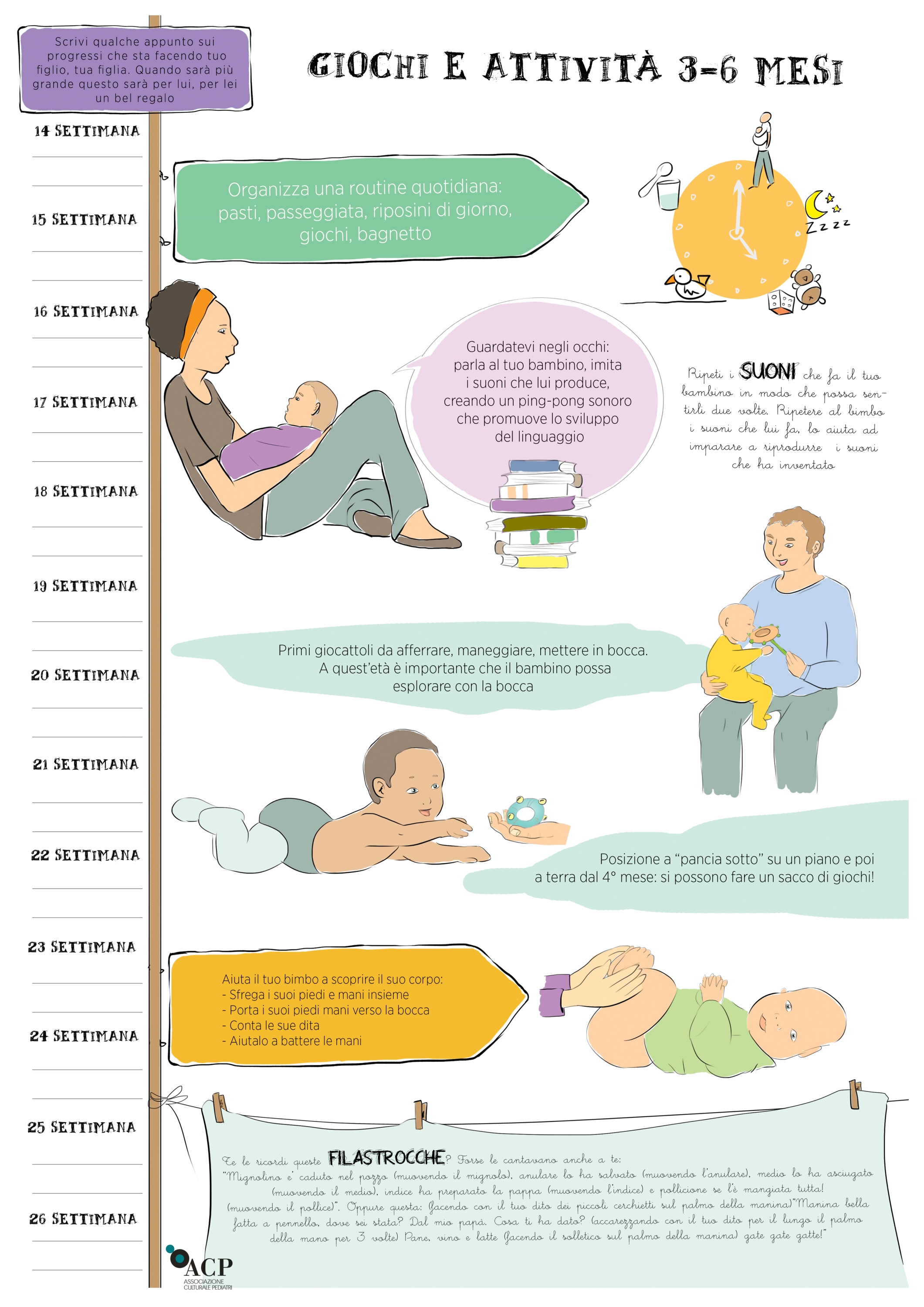

Supplement: Supplementary file 2 — Additional file 2. [file 13052_2020_886_MOESM2_ESM.zip › Appendix 2 poster 3-6 months.jpg]
