# Supplementary material for: Evaluation of an intervention aimed at supporting new parents: the Baby Newsletter project
Source: Ital J Pediatr. 2020 Sep 4;46:123. doi: 10.1186/s13052-020-00886-5 (PMC7487811; doi:10.1186/s13052-020-00886-5)
Supplement: Supplementary file 1 — Additional file 1. [file 13052_2020_886_MOESM1_ESM.zip › Appendix 1_translation_06_06_2020.doc]

ITALIAN:

Baby Newsletter – n.1 Nascita (p.2, Cosa significa essere un neonato)

Ho bisogno che qualcuno mi ami, mi nutra, mi tenga in braccio e giochi con me.

Mi piace sentire il calore, ma non troppo caldo. Attenzione a questi segnali: sudorazione, capelli bagnati, guance arrossate, respiro veloce.

Non mi piace il baccano e il frastuono

Mi piace essere sostenuto con delicatezza e gentilezza

Mi piace stare molto a contatto con chi mi vuole bene

Mi piace dormire

Spesso sono affamato, anche ogni 1-2 ore

Posso essere irritato e posso piangere tanto

La mia testa è grande e le mie braccia e gambe sono corte; quando mi abbracci, metti il tuo braccio sotto al mio collo per aiutarmi a reggere la testa.

Io tengo braccia e gambe flesse e le mie mani sono chiuse a pugno.

La mia pelle si può squamare

ENGLISH:

# Baby Newsletter –n.1 Birth (page 2 - what it means to be a newborn)

I need someone to love me, feed me, hold me, and play with me.

I like to feel warm, but not too hot. Watch out for these signs: sweating, damp hair, red cheeks, fast breathing.

I don't like noise.

I like to be held gently and carefully.

I like being very close to those who love me.

I like sleeping

I am often hungry, even every 1-2 hours

I can be fussy and I can cry a lot

My head is large and my arms and legs are short; when you hold me, put your arm under my neck to help me hold my head up.

I keep my arms and legs flexed and my hands closed in fist.

My skin can be scaly or peeling.
