# Supplementary material for: Evaluation of an intervention aimed at supporting new parents: the Baby Newsletter project
Source: Ital J Pediatr. 2020 Sep 4;46:123. doi: 10.1186/s13052-020-00886-5 (PMC7487811; doi:10.1186/s13052-020-00886-5)
Supplement: Supplementary file 2 — Additional file 2. [file 13052_2020_886_MOESM2_ESM.zip › Appendix 2_translation_06_06_2020.doc]

# Babynewsletter – poster giochi e attività 3-6 mesi

Scrivi qualche appunto sui progressi che sta facendo tuo figlio, tua figlia. Quando sarà più grande questo sarà per lui, per lei un bel regalo.

Organizza una routine quotidiana: pasti, passeggiata, riposini di giorno, giochi, bagnetto.

Guardatevi negli occhi: parla al tuo bambino, imita i suoni che lui produce, creando un ping-pong sonoro che promuove lo sviluppo del linguaggio

Ripeti i suoni che fa il bambino in modo che possa sentirli due volte. Ripetere al bimbo i suoni che lui fa, lo aiuta a imparare a riprodure i suoni che ha inventato.

Primi giocattoli da afferrare, maneggiare, mettere in bocca. A quest'età è importante che il bambino possa esplorare con la bocca

Posizione a "pancia sotto" su un piano e poi a terra dal 4° mese: si possono fare un sacco di giochi.

Aiuta il bambino a scoprire il suo corpo:

sfrega i suoi piedi e mani insieme, porta i suoi piedi o le suae mani verso la bocca, conta le sue dita, aiutalo a battere le mani

Canta le filastrocche che cantavano anche a te (segue una filastrocca italiana)

## Baby Newsletter – play and activities 3-6 months

Write some notes on the progress your baby is making. When he/ she gets older, this will be a nice gift.

Organize a daily routine: meals, walks, naps, games, bathtime.

Look into each other’s eyes: talk to your baby, imitate the sounds he/ she produces, creating conversational turns with cooing sounds and vocalization that promotes the development of language.

Repeat the sounds your baby makes so he/ she can hear them twice. Repeating back to your baby the sounds he/ she makes helps your baby learn to reproduce the sounds he/ she has invented.

At this age it is important that your baby can explore with his/her mouth: first toys to grab, handle, put in the mouth.

"Tummy time" position on the ground from the 4th month: a lot of games can be played in tummy time position.

Help your baby discover his/ her body: rub your baby’s feet and hands together, bring your baby’s feet or hands to his/ her mouth, count his/ her fingers, help your baby clap his/ her hands

Sing the nursery rhymes, including those your parents sang to you (an Italian nursery rhyme follows).
